# Hypertension in the association of triglyceride glucose‐body mass index with vascular brain injury in older adults: a population‐based MRI study

**DOI:** 10.1002/alz70856_101909

**Published:** 2025-12-25

**Authors:** Na Tian, Lin Song, Yun Liu, Jiafeng Wang, Cuicui Liu, Zhifeng Shang, Yifei Ren, Min Zhu, Heng Zhang, Keke Liu, Tingting Hou, Shi Tang, Lin Cong, Yongxiang Wang, Yifeng Du, Chengxuan Qiu

**Affiliations:** ^1^ Key Laboratory of Endocrine Glucose & Lipids Metabolism and Brain Aging, Ministry of Education; Department of Neurology, Shandong Provincial Hospital Affiliated to Shandong First Medical University, Jinan, Shandong, China; ^2^ Shandong Provincial Hospital Affiliated to Shandong First Medical University, Jinan, Shandong, China; ^3^ Medical Science and Technology Innovation Center, Shandong First Medical University & Shandong Academy of Medical Sciences, Jinan, Shandong, China; ^4^ Shandong Provincial Hospital affiliated to Shandong First Medical University, Jinan, Shandong, China; ^5^ Department of Neurology, Shandong Provincial Hospital, Shandong University, Jinan, Shandong, China; ^6^ Department of Neurology, Shandong Provincial Hospital Affiliated to Shandong First Medical University, Jinan, Shandong, China; ^7^ Department of Neurology, Shandong Provincial Hospital affiliated to Shandong First Medical University, Jinan, Shandong, P.R. China., Jinan, China; ^8^ Shandong Provincial Hospital Affiliated to Shandong First Medical University, Jinan, China; ^9^ Aging Research Center, Department of Neurobiology, Care Sciences and Society, Karolinska Institute‐Stockholm University, Solna, Sweden

## Abstract

**Background:**

Insulin resistance (IR) and hypertension, two well‐known vascular risk factors, have been closely linked to vascular brain injury. Evidence has suggested a causal relationship of IR to hypertension. However, population‐based studies have rarely explored the associations of the triglyceride glucose‐body mass index (TyG‐BMI), a surrogate marker of insulin resistance, with MRI markers of vascular brain injury, and the role of hypertension in their associations.

**Method:**

This population‐based study included 1268 participants (age ≥60 years; 58.60% women) from the MIND‐China MRI sub‐study (2018‐2020). White matter hyperintensities (WMH), enlarged perivascular spaces (EPVS), cerebral microbleeds (CMBs), and lacunes were assessed following the STRIVE‐1 criteria. TyG‐BMI index was calculated as ln(fasting triglyceride(mg/dL)×fasting glucose(mg/dL)/2)×BMI. Data were analyzed using general linear, logistic, and mediation models.

**Result:**

A higher TyG‐BMI index was significantly associated with greater volumes of total WMH (multivariable‐adjusted β=0.018; 95% confidence interval=0.002‐0.024), periventricular WMH (0.017; 0.001‐0.033), and deep WMH (0.010; 0.002‐0.019), and an increased likelihood of WMH burden (multivariable‐adjusted odds ratio=1.15; 95% confidence interval=1.02‐1.29), but not with EPVS, CMBs or lacunes (*p* >0.05). In the mediation analysis, hypertension significantly mediated around 42.86% of the association of TyG‐BMI index with WMH volume. The observed associations between TyG‐BMI index and WMH as well as the mediation by hypertension remained significant among non‐diabetic participants.

**Conclusion:**

IR, as indicated by high TyG‐BMI index, is associated with vascular brain injury in older adults, even among those free of diabetes, in which their associations are largely mediated by hypertension.

